# Methylglyoxal induces p53 activation and inhibits mTORC1 in human umbilical vein endothelial cells

**DOI:** 10.1038/s41598-021-87561-9

**Published:** 2021-04-13

**Authors:** Xinmiao Zhang, Angelica Rodriguez-Niño, Diego O. Pastene, Prama Pallavi, Jacob van den Born, Stephan J. L. Bakker, Bernhard K. Krämer, Benito A. Yard

**Affiliations:** 1 Department of Nephrology, Endocrinology and Rheumatology, Fifth Department of Medicine, University Hospital Mannheim, Heidelberg University, Theodor-Kutzer-Ufer 1-3, 68167 Mannheim, Germany; 2 Surgical Department, University Hospital Mannheim, Heidelberg University, Mannheim, Germany; 3 Department of Internal Medicine, University Medical Center Groningen, University of Groningen, Groningen, The Netherlands; 4 European Center for Angioscience, Medical Faculty Mannheim, Heidelberg University, Mannheim, Germany

**Keywords:** Cell biology, Molecular biology, Endocrinology, Molecular medicine

## Abstract

Methylglyoxal (MGO), a precursor of advanced glycation end products (AGEs), is regarded as a pivotal mediator of vascular damage in patients with diabetes. We have previously reported that MGO induces transcriptional changes compatible with p53 activation in cultured human endothelial cells. To further substantiate this finding and to explore the underlying mechanisms and possible consequences of p53 activation, we aimed (1) to provide direct evidence for p53 activation in MGO-treated human umbilical vein endothelial cells (HUVECs), (2) to assess putative mechanisms by which this occurs, (3) to analyze down-stream effects on mTOR and autophagy pathways, and (4) to assess the potential benefit of carnosine herein. Exposure of HUVECs to 800 µM of MGO for 5 h induced p53 phosphorylation. This was paralleled by an increase in TUNEL and γ-H2AX positive cells, indicative for DNA damage. Compatible with p53 activation, MGO treatment resulted in cell cycle arrest, inhibition of mTORC1 and induction of autophagy. Carnosine co-treatment did not counteract MGO-driven effects. In conclusion, our results demonstrate that MGO elicits DNA damage and p53 activation in HUVECs, resulting in modulation of downstream pathways, e.g. mTORC1.

## Introduction

The prevalence of diabetes has gradually increased worldwide, already affecting in 2019 9.3% (463 million people) of the global population^1^. Because of its high associated morbidity, diabetes poses a large social and healthcare burden^2^. Patients with diabetes have an increased risk to suffer from macro- and/or micro-vascular complications^3,4^. These complications may progress to more severe diseases, e.g. accelerated atherosclerosis, end-stage renal failure, peripheral nerve dysfunction and acquired blindness, resulting in high disability and mortality among diabetic patients^5,6^.

Emerging evidence indicates that the accumulation of advanced glycation end-products (AGEs) may contribute to the pathogenesis of vascular damage in diabetes mellitus^7–9^. Indeed the levels of AGEs are increased in serum, renal tissue and retinal vessels of diabetic patients^10,11^. Methylglyoxal (MGO), a highly reactive dicarbonyl metabolite and precursor of AGEs, is under normal conditions efficiently detoxified by the glyoxalase-1(Glo1) and glyoxalase-2 (Glo2) enzymes^12^. This will prevent its reaction with protein residues and nucleic acids, thereby limiting the formation and accumulation of stable MGO-derived AGEs and DNA adducts^8,13^. In addition, MGO can stimulate oxidative stress by the induction of reactive oxygen species (ROS) production^14,15^.

In in vitro or in in vivo models, elevated levels of MGO that are not caused by hyperglycemia result in pathological changes similar to those observed in patients with diabetes. These pathological changes include vascular damage, insulin resistance and low grade inflammation^16–18^. In line with this, one study has reported that exogenously administered MGO to type 2 diabetic rats aggravates endothelial dysfunction and oxidative stress^19^. Furthermore, downregulation of Glo1 results in high endogenous levels of MGO causing vascular inflammation and dysfunction of cultured endothelial cells^20^. In contrast, overexpression of Glo1 resulted in reduced diabetes-induced formation of AGEs in mesenteric arteries of diabetic rats and improvement of endothelial dysfunction^20^.

Although the mechanisms underlying MGO-associated endothelial dysfunction are not well understood, compelling evidence points to an upregulation of apoptosis-mediated mechanisms. In a previous study, we demonstrated that in human umbilical vein endothelial cells (HUVECs) MGO treatment strongly regulates cell cycle-associated genes, among which cyclin dependent kinase 1(CDK1) and cyclin B2 (CCNB2) were found to be strongly affected. These transcriptional changes are compatible with cell cycle arrest and activation of the p53 pathway^21^. The transcription factor p53 is sensitive to DNA damage and cellular stress and plays a critical role in regulating cell-growth signaling pathways^22^. In addition, p53 activation is associated with the development of metabolic disorders and diabetes, including pancreatic dysfunction, impaired insulin sensitivity and negative regulation of glycolysis^23,24^. MGO may cause glycation of nucleosomal protein or induce DNA strand breaks that favor p53 activation^25,26^. Whether this indeed occurs in endothelial cells is currently not known. In keeping with our previous study which suggested MGO might activate p53, in the present study we sought to provide formal proof that p53 is indeed activated in cultured endothelial cells by MGO in an attempt to better understand the biological effect of MGO on cell growth and other p53-regulated pathways, including the mTORC1 and autophagy pathways. We also assessed whether carnosine (Car), a histidine containing dipeptide with scavenging and antioxidant properties, is capable of counteracting MGO-driven effects.

## Results

### Cell morphology and viability are affected by MGO

**Figure 1:**
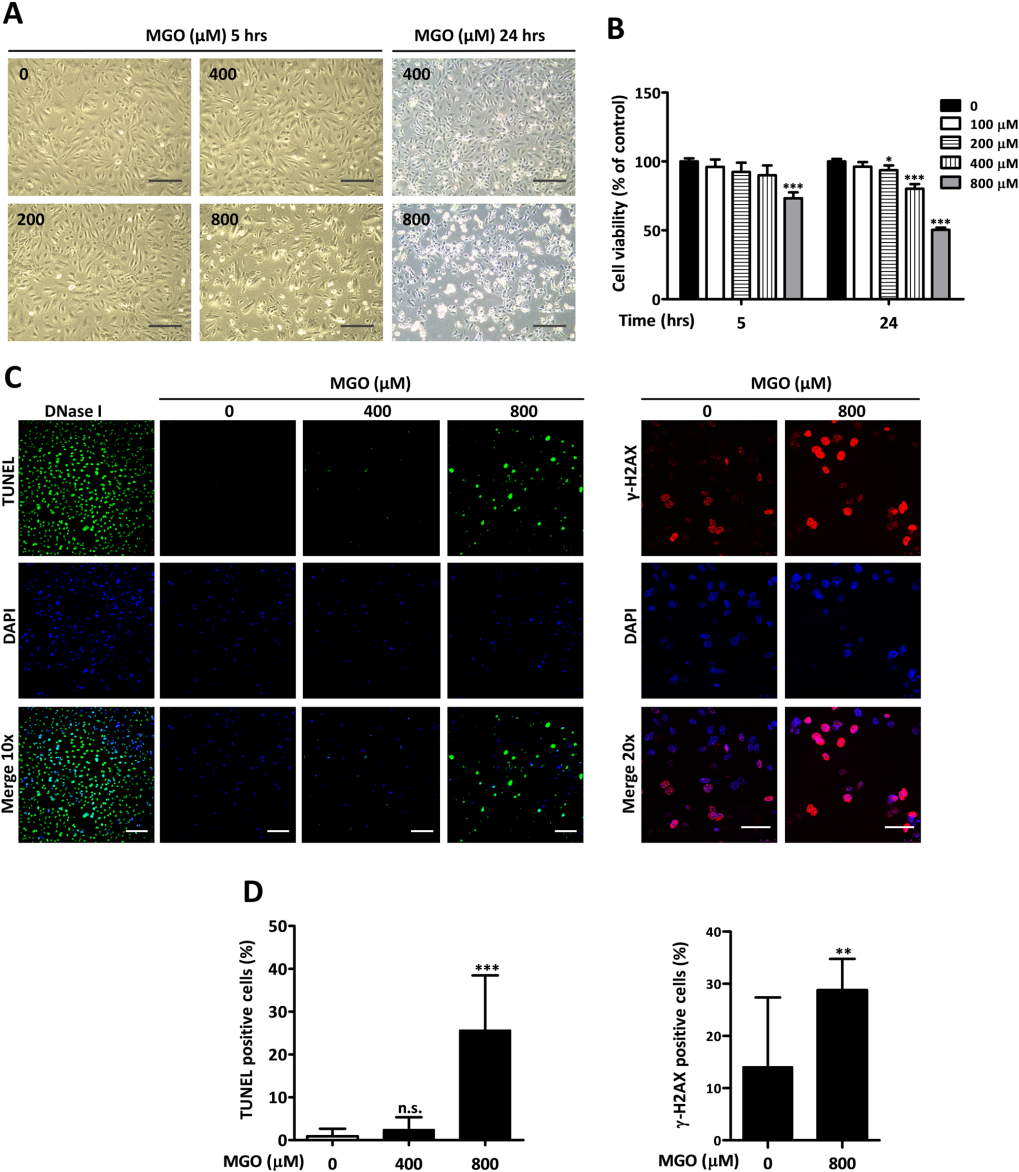
Cell morphology and viability are affected by MGO. (**A**) HUVECs were stimulated with different concentrations of MGO. The cells were inspected by phase-contrast microscopy after 5 and 24 h. Changes in cell morphology, i.e. cell detachment, deformation and shrinkage, were already observed after 5 h at the highest MGO concentration. Original magnification: 10x, scale bar represents 250 µm. (**B**) Cell viability was assessed by MTT assay after 5 h (panel to the left) and 24 h (panel to the right) post MGO treatment. The results are expressed as mean % of viable cells ± SD of 5 independent experiments. *p < 0.05 and ***p < 0.001 vs. no MGO. (**C**) DNA double stand breaks (panel to the left) and expression of γ-H2AX (panel to the right) were assessed by TUNEL and immunofluorescence respectively. DAPI was used to stain the nucleus. DNase I served as a positive control. Cells were visualized under fluorescence microscope. Original magnification: 10 × for TUNEL, scale bar represents 150 µm; 20 × for γ-H2AX, scale bar represents 50 µm. At least 3 independent experiments were performed. (**D**) The quantification of positive TUNEL and γ-H2AX cells are shown as mean % of positive cells ± SD. **p < 0.01 and ***p < 0.001 vs. no MGO, n.s. represents non-significant.

In order to investigate the effects of MGO on endothelial cells, we initially exposed HUVECs to a wide range of MGO concentrations (0–800 µM) and studied cell morphology microscopically at different time points. Already 5 h after MGO exposure cell morphology changed at 800 µM of MGO, which became more prominent after 24 h of exposure. No evident changes were observed when endothelial cells were exposed to MGO concentrations below 400 µM (Fig. 1A). MTT assays were subsequently performed to explore if the different concentrations of MGO affected cell viability. As shown in Fig. 1B, the results of the MTT assays were roughly equivalent to the microscopic observation with a cell viability of approximately 50% or less after 24 h of exposure using 800 µM of MGO. TUNEL assays were performed to assess if cell toxicity was associated with DNA damage (Fig. 1C, to the left). While incubation of cells with DNase I clearly resulted in TUNEL positivity in all cells, in endothelial cells exposed to 800 µM of MGO approximately 25% of cells were TUNEL positive after 5 h (Fig. 1D, to the left). In line with this, histone H2AX phosphorylation, i.e. γ-H2AX, increased (Fig. 1C, to the right) and was present in approximately 28% of endothelial cells that were exposed for 5 h to 800 µM of MGO (Fig. 1D, to the right).

### p53 activation by MGO

**Figure 2:**
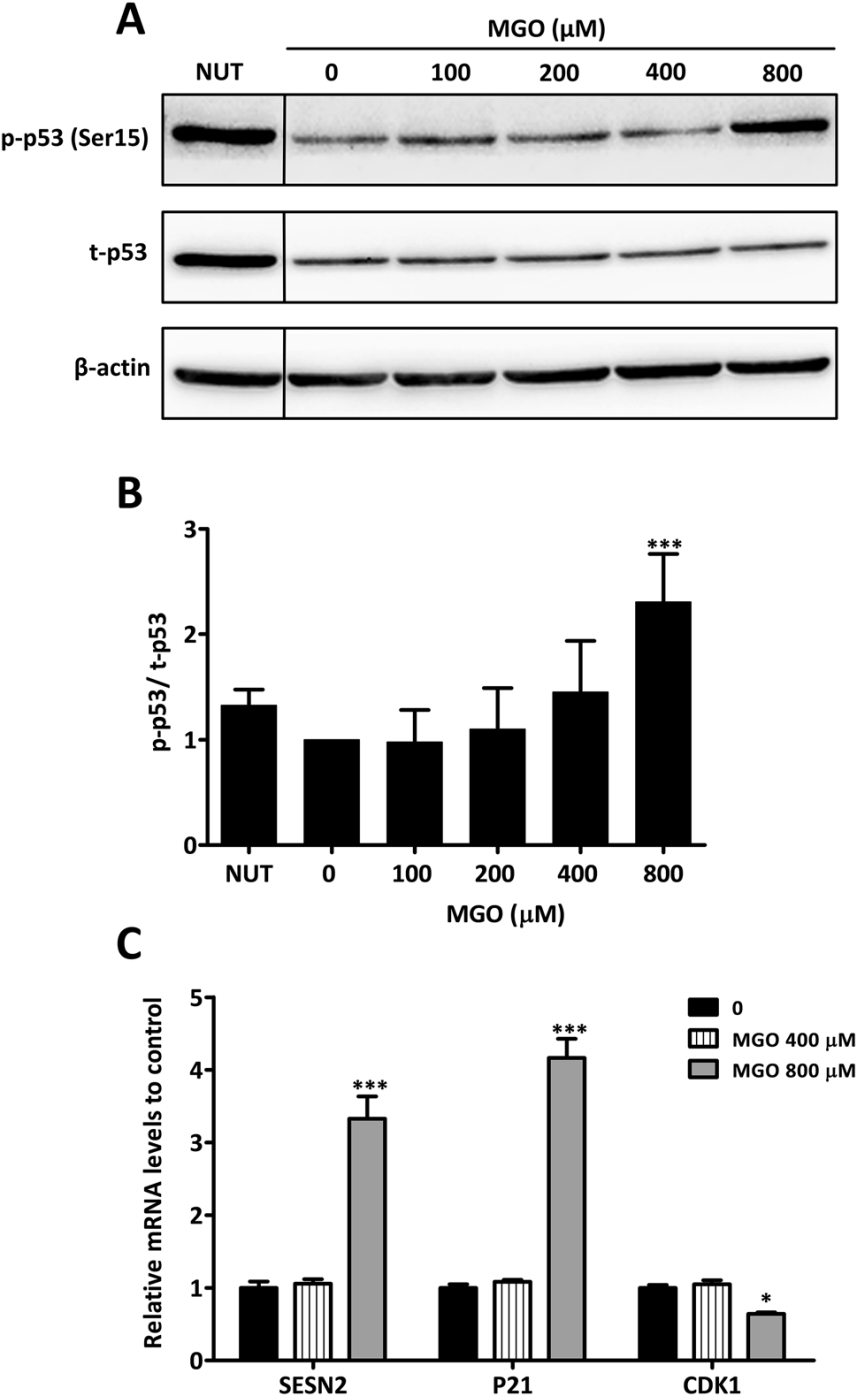
Activation of p53 by MGO. (**A**) HUVECs were stimulated for 5 h with different concentrations of MGO. Nutlin-3a (NUT) (10 µM) was used as a positive control. The cell lysates were processed for gel-electrophoresis and western blotting using anti-phosphorylated p53 (p-p53) and anti-total p53 (t-p53) antibodies. The results of a representative experiment are shown. Displayed are the cropped blots. The NUT lanes are non-contiguous parts from the same gel and indicated by a black vertical line. Original blots are provided in Supplementary Fig. S2. (**B**) Quantification of 3 independent experiments was performed by densitometry. The results are expressed as mean p-p53/ t-p53 ratio ± SD. (**C**) HUVECs were treated for 5 h with different MGO concentrations. Hereafter, SESN2, P21 and CDK1 mRNA levels were assessed by qRT-PCR and normalized for β-actin. The results of 3 independent experiments are expressed as mean fold change ± SD relative to untreated HUVECs. *p < 0.05 and ***p < 0.001 vs. no MGO.

Even though only a relative small percentages of cells was positive TUNEL or γ-H2AX respectively, DNA damage per se may lead to p53 activation and thus to initiation of a DNA repair process^27^. Because phosphorylation of serine 15 (Ser15) in the p53 protein is a primary target of the DNA damage response, we assessed p53 Ser15 phosphorylation after MGO exposure. Nutlin-3a (NUT-3), blocking MDM2-mediated degradation of p53, was used as representative of p53 activators to demonstrate specificity for the total p53 (t-p53) and phosphorylated p53 (p-p53) binding antibodies. Endothelial cells that were exposed for 5 h to high MGO concentration (800 µM) displayed an increase of Ser15 phosphorylation on p53 (Fig. 2A,B). Because for longer exposure time cell viability decreased to 50% or less in some experiments at 800 µM of MGO, western blotting was only performed for the 5 h exposure time. To further substantiate the finding that p53 was activated at 800 µM of MGO, we assessed mRNA expression of downstream targets that are transcriptionally regulated by p53. It was found that mRNA expression of SESN2, p21 (CDKN1A) and CDK1 was changed following 5 h of stimulation with 800 µM of MGO. While SESN2 (3.3 fold) and p21 (4.2 fold) mRNA levels increased, the mRNA expression of CDK1 decreased (0.6 fold) compared to untreated control (Fig. 2C).

### MGO inhibits the mTOR pathway

**Figure 3:**
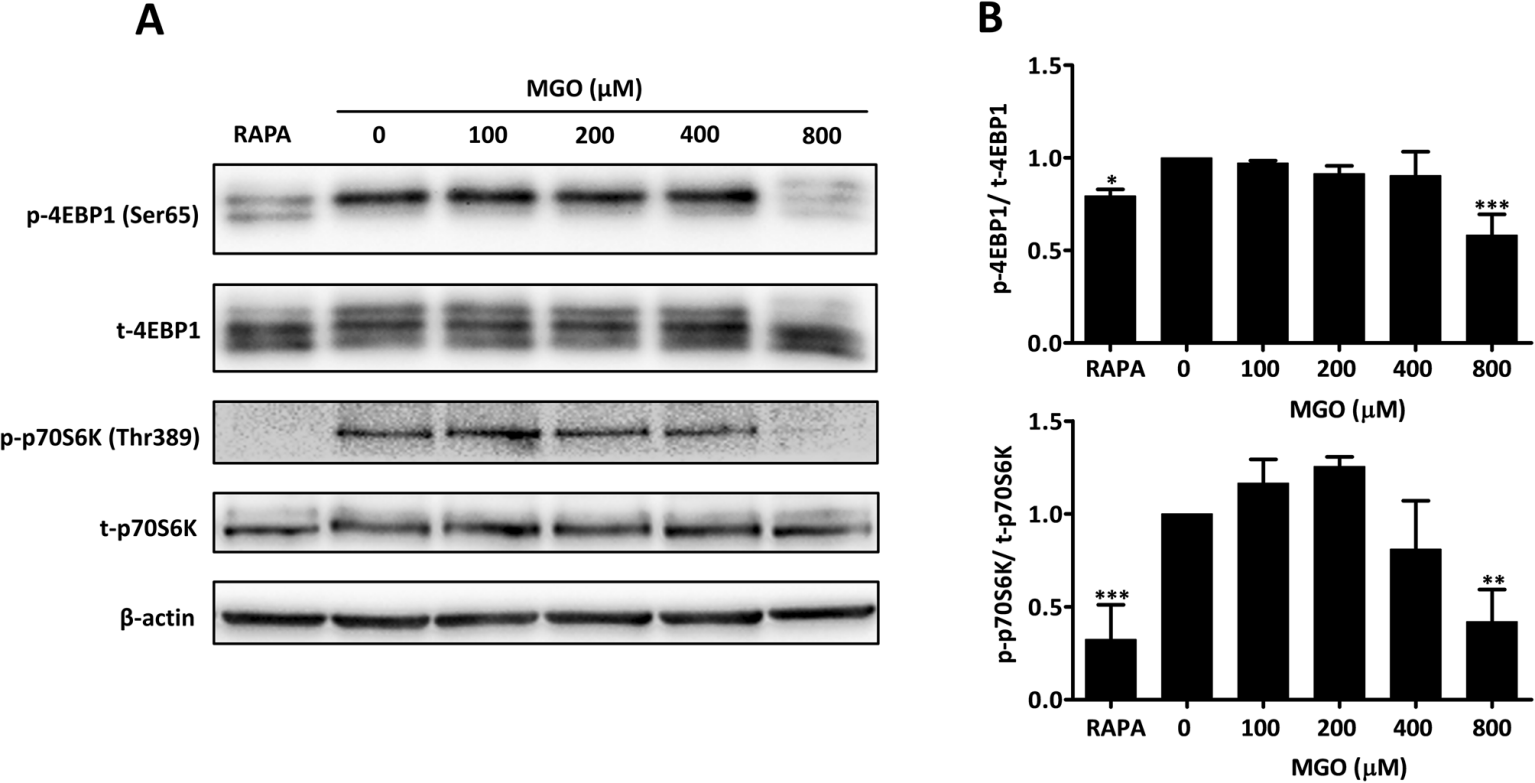
Inhibition of mTORC1 by MGO. (**A**) HUVECs were treated for 5 h with the indicated concentrations of MGO or rapamycin (RAPA) (5 µM). Hereafter, cell lysates were processed for gel-electrophoresis and western blotting using anti-phosphorylated (p-) and total (t-) p70S6K or 4EBP1. Anti-β-actin was used to demonstrate equal loading. The results of a representative experiment are shown. Displayed are the cropped blots and original blots are provided in Supplementary Fig. S3. (**B**) Quantification of 4 independent experiments was performed by densitometry. The results are expressed as mean p-/t-4EBP1 and p-/t-p70S6K ± SD. *p < 0.05, **p < 0.01 and ***p < 0.001 vs. no MGO.

**Figure 4:**
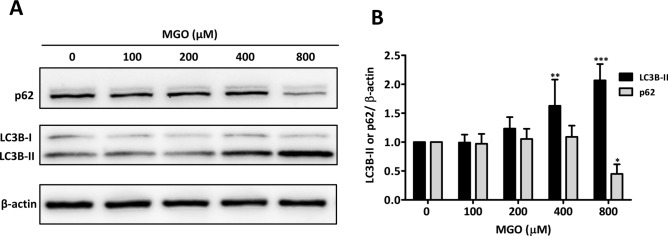
Induction of autophagy by MGO. (**A**) The cell lysates as described in Fig. 3 were also used to assess the expression of p62 and LC3B as markers for autophagy. The results of a representative experiment are shown. Displayed are the cropped blots and original blots are provided in Supplementary Fig. S4. (**B**) Quantification of p62 and LC3B-II was performed by densitometry as measure for autophagy. The results of 4 independent experiments are expressed as mean LC3B-II or p62/β-actin ratio ± SD. *p < 0.05, **p < 0.01 and ***p < 0.001 vs. no MGO.

The p53 pathway restrains mTORC1 activation under conditions of genotoxic stress through the transcription of sestrins (SESN1 and SESN2). We therefore assessed if 800 µM of MGO affected downstream targets of mTORC1 activation, including 4EBP1 and p70S6K. Similar to rapamycin (Rapa) (5 µM), MGO inhibited the phosphorylation of both target proteins suggesting inhibition of mTORC1 (Fig. 3A,B). Because inhibition of mTORC1 may increase autophagy, the expression of p62 and LC3B was studied. In line with increased autophagy, it was found that p62 expression decreased with a concomitant increase in LC3B-II expression (Fig. 4A,B).

### Carnosine does not ameliorate MGO driven-p53 activation or mTORC1 inhibition

**Figure 5:**
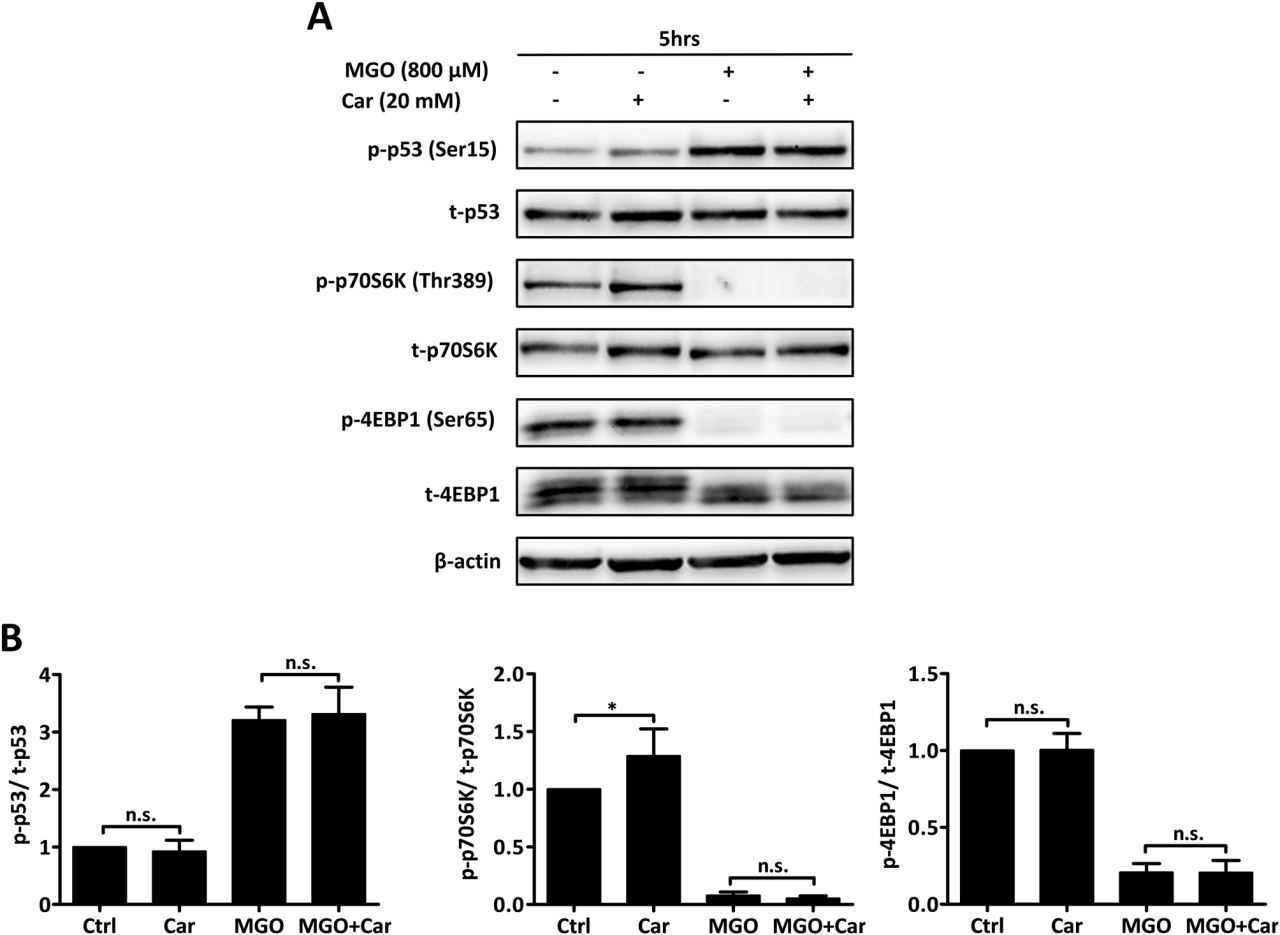
Carnosine does not ameliorate MGO-induced p53 activation or mTORC1 inhibition. (**A**) HUVECs were stimulated for 5 h with 800 µM of MGO. Stimulation occurred either in the presence or absence of 20 mM carnosine (Car). Hereafter, the cell lysates were processed for gel-electrophoresis and western blotting using anti-phosphorylated (p-) and anti-total (t-) p53, p70S6K and 4EBP1. The results of a representative experiment are shown. Displayed are the cropped blots and original blots are provided in Supplementary Fig. S5. (**B**) Quantification of 3 independent experiments was performed by densitometry. The results are expressed as mean p-/t-p53, p-/t-p70S6K and p-/t-4EBP1 ratio ± SD. *p < 0.05 vs. no MGO, n.s. represents non-significant.

**Figure 6:**
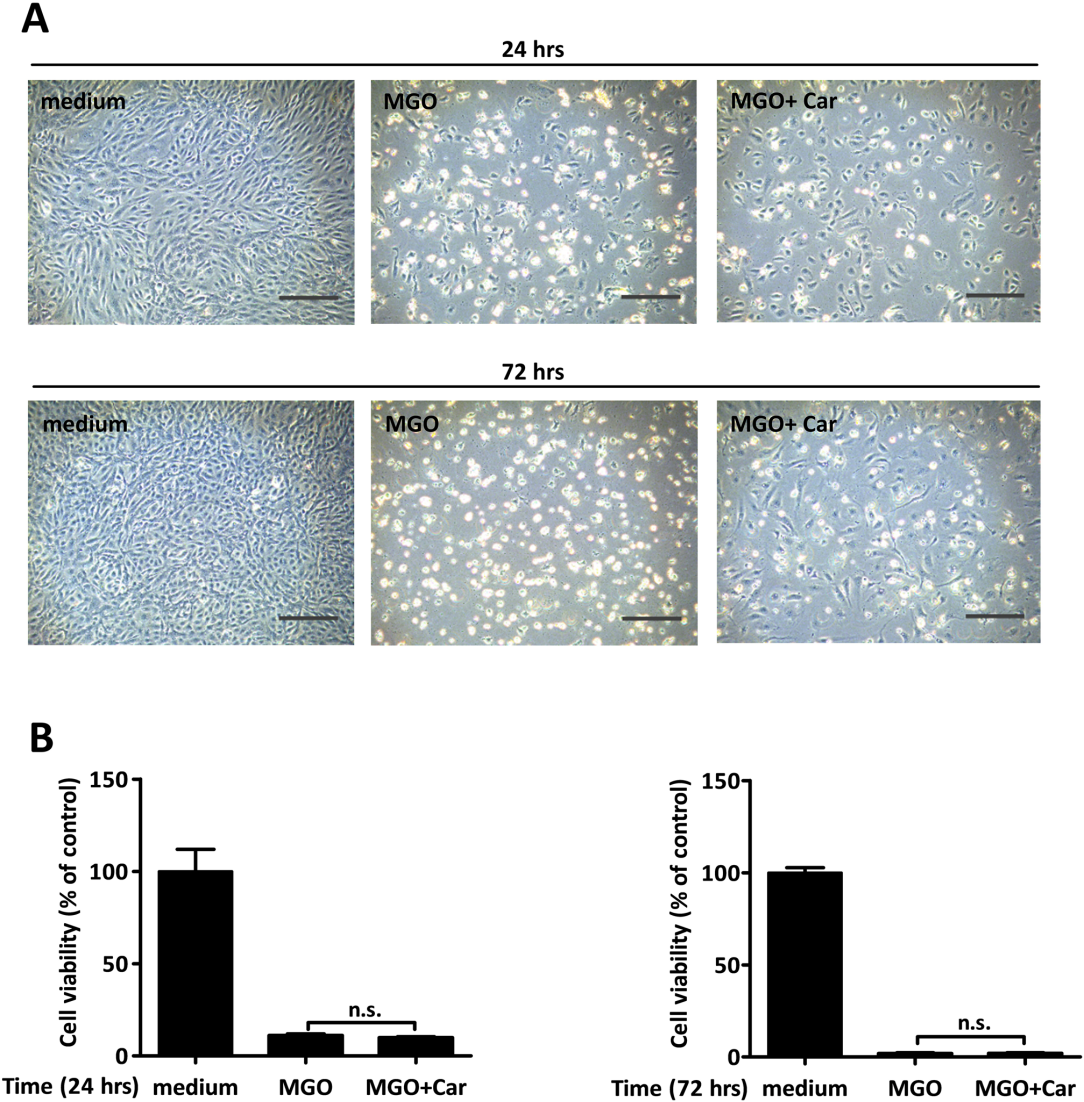
Carnosine does not alleviate MGO-mediated cell toxicity. HUVECs were challenged with 800 µM of MGO for a total period of 72 h in the absence or presence of 20 mM carnosine (Car). (**A**) Morphological changes were monitored by phase contrast microscopy at 24 h (upper panel) and 72 h (lower panel). Original magnification: 10x, scale bar represents 250 µm. (**B**) MTT assay was employed to assess cell viability at 24 h (figure to the left) and 72 h (figure to the right). The results are expressed as mean % of viable cells ± SD of 5 independent experiments.

In keeping with carnosine’s ability to scavenge reactive carbonyl species^28^, we tested if the presence of carnosine would ameliorate MGO-driven effects. To this end, endothelial cells were challenged with 800 µM of MGO in the presence or absence of 20 mM of l-carnosine (Car) and activation of p53 as well as that of the downstream targets 4EBP1 and p70S6K was assessed. Carnosine alone slightly but significantly increased the expression of phosphorylated p70S6K, but was not able to ameliorate MGO driven-p53 activation or inhibition of mTORC1 (Fig. 5A,B). The addition of carnosine to MGO challenged endothelial cells did not ameliorate endothelial damage as assessed in MTT assays (Fig. 6B), albeit that in some experiments microscopic evaluation suggested recovery of cells at later time-points (Fig. 6A). This was unlikely due to carnosine per se as it was also observed in single experiments when cells were challenged with MGO only.

### Non-enzymatic protein glycation and reactive oxygen species (ROS)

**Figure 7:**
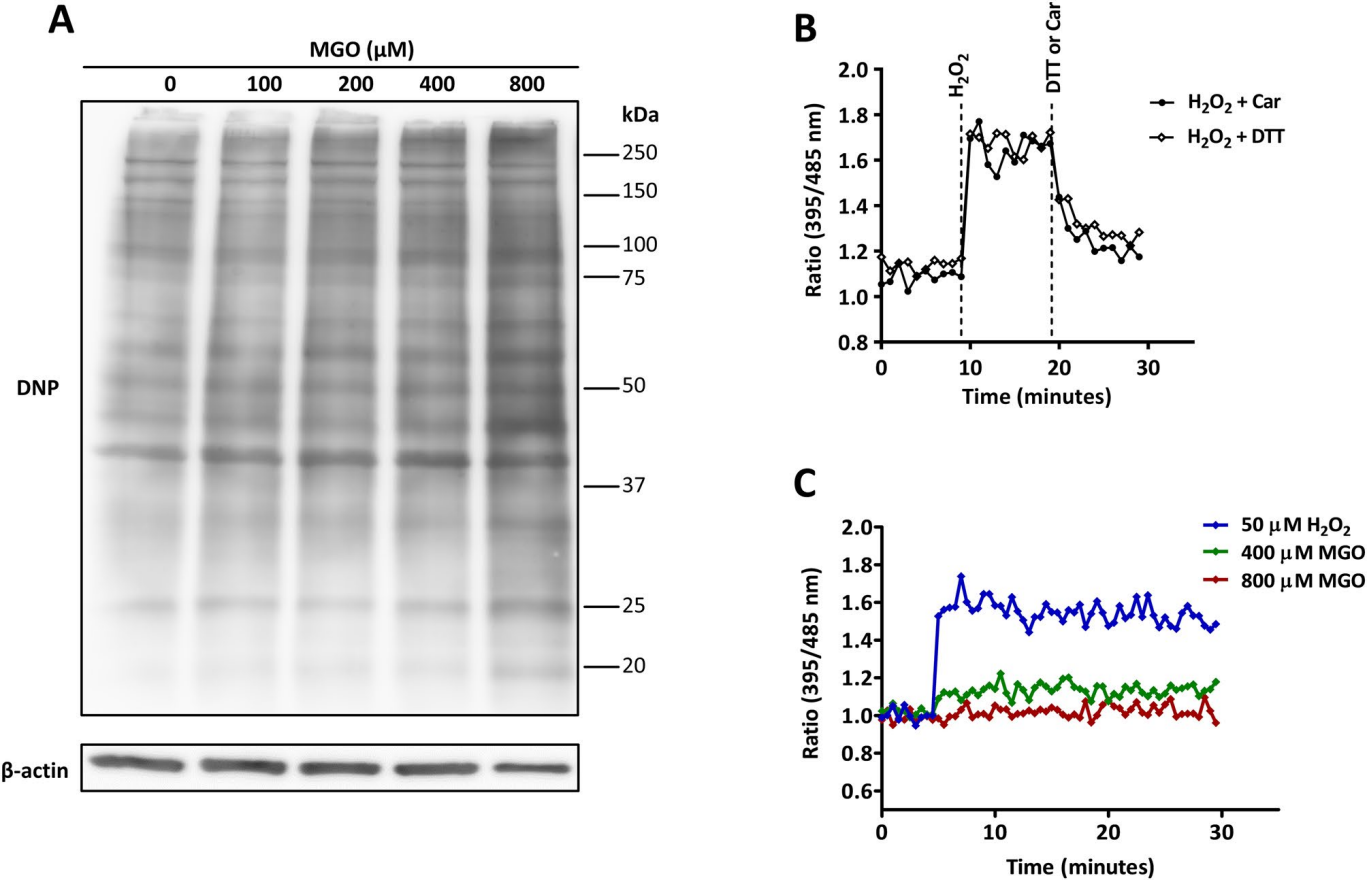
MGO causes protein glycation but not oxidative stress in HUVECs. (**A**) HUVECs were treated with varying concentrations of MGO for 5 h. Hereafter, DNP-derivatized cell lysates were subjected to immunoblotting to assess protein carbonylation. β-actin as loading control was made using the same amount of samples processed by SDS–polyacrylamide gel-electrophoresis. Displayed are the cropped blots and full-length blots are provided in Supplementary Fig. S6. **(B)** HUVECs expressing roGFP3 were excited at 395 nm and at 485 nm, and the ratio of emission at 520 nm was calculated. After 10 min, cells were challenged with 50 µM H_2_O_2_ followed by the addition of 500 µM DTT or 20 mM carnosine (Car). Changes in the redox response were monitored over a total period of 30 min. **(C)** HUVECs expressing roGFP3-Grx were challenged with 50 µM H_2_O_2_ or different concentrations of MGO and changes in the emission ratio at 520 nm after excitation at 395 and 485 nm were monitored.

It has been suggested that MGO can yield advanced glycation end products (AGEs) via non-enzymatic glycation of proteins, and this in turn increases ROS production^29,30^. Hence, apart from DNA damage, oxidative stress might be an alternative mechanism of MGO’s toxicity. We therefore first determined the degree of protein carbonylation in response to different concentrations of MGO. As compared to untreated endothelial cells, 800 µM of MGO clearly caused increased protein carbonylation (Fig. 7A). Subsequently, we made use of the roGFP redox sensor to assess if increased carbonylation resulted in oxidative stress. In roGFP transduced HUVECs, H_2_O_2_ dose dependently increased the fluorescence ratio at 395/485 nm (Supplementary Fig. S1), which returned to baseline after addition of DTT or carnosine (Fig. 7B). While the intracellular redox sensor was able to respond to these exogenously added oxidizing or reducing agents, none of the MGO concentrations increased the excitation fluorescence ratio (395/485 nm) emitted at 528 nm (Fig. 7C). Therefore, MGO can lead to protein carbonylation, albeit it did not affect GSH/ GSSG (reduced/oxidized glutathione) levels detected by roGFP sensor.

## Discussion

We previously demonstrated that the gene expression profile of cultured endothelial cells is strongly altered upon MGO exposure. Gene ontology (GO) enrichment analysis revealed that these changes are, amongst others, compatible with activation of the p53 pathway^21^. In keeping with this and because emerging evidence suggest a role for p53 in the development of metabolic diseases^23,24^, the present study was conducted to further substantiate our initial findings on MGO mediated p53 activation and to assess the consequences hereof on downstream pathways such as mTORC1. Our findings indicate that 800 µM of MGO is associated with DNA damage, is capable of activating p53, regulates cell cycle dependent genes and inhibits mTORC1. We also demonstrate that MGO activates autophagy, possibly as the result of mTORC1 inhibition.

The tumor suppressor p53 acts as a transcription factor, regulating a plethora of signaling molecules. Its role has been discussed as a double-edged sword that relays a wide range of pro-survival signals early in the DNA damage response but that shifts to apoptosis or senescence if damage continues^31^. High MGO concentrations likely cause DNA damage as evidenced by TUNEL and γ-H2AX expression (Fig. 1C), which is generally increased under genotoxic conditions^25^. Our data are compatible with the findings of Thornalley et al*.*^26^ demonstrating that MGO causes DNA glycation and formation of nucleotide adducts in HL-60 cells. This also explains our previous findings on gene set enrichment for the GO term “cellular response to DNA damage stimulus” when endothelial cells were stimulated with 800 µM of MGO^21^. Hence, our findings indicate that MGO to some extent might trigger a DNA damage repair response involving p53 activation and cell-cycle arrest and may cause cell toxicity if damage is beyond repair^32^.

Activation of p53 is initiated by phosphorylation on Serine 15 (Ser15) followed by additional phosphorylation events on other residues^33,34^. Thus, assessment of p53 (Ser15) phosphorylation is a conceivable method for evaluation of p53 activation. Indeed, 800 µM of MGO caused almost 2.3-fold increase in p53 (Ser15) phosphorylation already after 5 h of MGO exposure (Fig. 2A,B). Nutlin-3a was included in a series of experiments as a positive control. Nutlin-3a not only stabilizes p53 by disrupting the p53-MDM2 interaction, but also induces p53 phosphorylation in the DNA damage response^35,36^. When compared to Nutlin-3a, MGO induced more phosphorylation but did not evidently affect total p53 expression (Fig. 2A,B). While a previous study suggested that MGO upregulates p53 expression in endothelial cells^37^, our own data demonstrate that MGO is more likely affecting p53 activity through its increased phosphorylation.

Activation of p53 subsequently induces or represses the transcription of multiple downstream effector genes to regulate cellular programs^38,39^. We have assessed p53 downstream targets related to cell cycle progression, i.e. p21 and CDK1, and related to regulation of the mTOR pathway, i.e. SESN2. While SESN2 and p21 mRNA levels were three to fourfold upregulated, CDK1 mRNA was strongly diminished following MGO treatment (Fig. 2C). Thus, our findings indicate that MGO-induced p53 activation up- or down-regulates its transcriptional targets, which may result in cell cycle arrest and inhibition of mTORC1.

Being a DNA damage-inducible protein, SESN2 plays a critical role in the response to genotoxic stress^40^. It has been reported that SESN2 inhibits mTORC1 signaling by activating AMPK signaling^41,42^. Under genotoxic conditions, inhibition of mTOR signaling may shift energy expenditure to make more energy available for DNA repair^43–45^. In keeping with our findings that MGO activates p53 and upregulates SESN2, we assessed if suppression of mTORC1 activity occurred by evaluating its downstream targets 4EBP1 and p70S6K. Similar to rapamycin (RAPA), a known suppressor of mTOR, both 4EBP1 (Ser65) and p70S6K (Thr389) phosphorylation were inhibited by 800 µM of MGO (Fig. 3A,B).

Autophagy is an evolutionarily conserved lysosome-dependent system of which its main function is to maintain cellular homeostasis by removal of dysfunctional proteins and organelles^46^. Similar as shown for retinal pigment epithelial and HT22 nerve cells^47,48^, our current study demonstrates that MGO induces autophagy in cultured endothelial cells (Fig. 4A,B). It is conceivable that induction of autophagy occurs through the inhibition of mTORC1^45,49^, albeit that in tumor cells autophagy can be induced by AMPK activation without inhibition of mTORC1 or the phosphorylation of its substrate p70S6K^50^.

Carnosine (Car) is a histidine-containing dipeptide that can counteract carbonyl and oxidative stress in vitro and in vivo^51–53^. The ability of carnosine to reduce carbonyl stress strongly depends on its ability to quench the different reactive carbonyl species (RCS). While adducts between a variety of such RCS and carnosine have been observed in a variety of studies, thus far adducts between MGO and carnosine have not been identified. This therefore explains why carnosine was neither able to alleviate MGO-induced p53 activation nor to rescue MGO-associated mTOR inhibition (Fig. 5). These findings are in good agreement with the study of Weigand et al*.* that exogenous administration of carnosine did not mitigate MGO-mediated toxicity in cultured renal cells^54^. Indeed, it has been suggested that carnosine’s quenching activity is strong towards α and β unsaturated carbonyls, but rather poor for dicarbonyls such as MGO^55^.

It has been reported that MGO promotes oxidative stress through the generation of ROS, which in turn might change the intracellular reduction–oxidation (redox) potential. By making use of a genetically encoded GFP coupled to Glutaredoxin-1 (roGFP-Grx1) sensor in HUVECs, we tested to what extent MGO alters the intracellular redox milieu. This sensor allows the measurement of rapid alterations in the redox equilibrium based on the ability of GFP to become oxidized or reduced by changes in the reduced/oxidized glutathione/glutathione disulfide (GSH/GSSG) couple which act as a Grx-1 target protein^56^. While treatment with oxidizing and reducing agents, i.e. H_2_O_2_ and DTT, caused rapid changes in the emission ratio of roGFP-Grx1 (Fig. 7B), no changes hereof were found when HUVECs were stimulated with MGO (Fig. 7C). It therefore seems that MGO is not causing an acute burst of ROS as this would affect the intracellular GSH/GSSG redox buffer. Even though it is reported that MGO detoxification through the glyoxalase pathway is glutathione (GSH)-dependent^12^, it should be emphasized that there is no net change in GSH concentrations as GSH is recycled back^57^. Nonetheless, at low concentrations MGO may exert a pro-oxidative effect on endothelial cells when exposed for longer periods of time involving the upregulation of NOX4^58^.

Although the results presented in our study confirm and extend previous observations, there are a few limitations that need to be discussed. First, our study was designed based on previous Affymetrix data, solely corresponding to one cell type (HUVEC) ^21^. In keeping with the significant heterogeneity in endothelial morphology and function our findings therefore should not be generalized and transferred to other types of endothelial cells (e.g. microvascular) that are implicated in diabetic complications. Nonetheless, p53 activation by MGO has been reported by others^59^. Second, we are aware that the MGO concentrations used in this study by far exceed MGO concentrations reported in plasma of healthy individuals or patients with diabetes. Yet, the concentrations used in our study are well within the concentration range reported in other in vitro studies^47,48,58^ and similar to our previous study^21^.

In conclusion, our study demonstrates that high concentrations of MGO lead to DNA damage, which in turn activates p53 and subsequently inhibits mTORC1 in HUVECs. Because the concentrations used in our study by far exceed plasma concentration in diabetic patients, it would be prudent to take some caution in interpreting our data that MGO would have a similar action in vivo. Inasmuch as MGO concentrations in serum of diabetic patients may not suffice to activate p53 directly, in conjunction with other noxious signals it may potentially contribute to DNA damage. The role of p53 activation for diabetic complications has been illuminated in various diabetic models, e.g. in db/db and STZ-induced diabetic nephrology (DN) mice, whether this is a direct consequence of MGO remains to be addressed^60^.

## Methods

### Antibodies

Antibodies used in western blotting: rabbit-anti-phospho-p53 (Ser15) (Cell Signaling Technology, Danvers, USA); mouse-anti-p53 (Santa Cruz Biotechnology, Dallas, USA); rabbit-anti-phospho-p70S6K (Thr389) (Cell Signaling Technology); rabbit-anti-p70S6K (Cell Signaling Technology); mouse-anti-phospho-4EBP1 (Ser65) (Santa Cruz Biotechnology); rabbit-anti-4EBP1 (Cell Signaling Technology); mouse-anti-p62/ SQSTM1 (Santa Cruz Biotechnology); rabbit-anti-LC3B (Thermo Fisher Scientific, Waltham, USA); mouse-anti-β-actin (Santa Cruz Biotechnology); horseradish peroxidase (HRP) conjugated secondary antibodies (Santa Cruz Biotechnology).

### Cell culture and treatments

HUVECs were isolated from fresh umbilical cords obtained from Department of Obstetrics, University Medical Centre Mannheim with informed consent. The cells were cultured on 1% gelatin-coated flasks in endothelial growth cell growth medium (Provitro, Berlin, Germany) with essential supplements, and maintained at 37 °C, 95% relative humidity and 5% CO_2_. Confluent monolayers were passaged by TrypLE Select Enzyme (Thermo Fisher Scientific) and cells on passage 2–5 were used to conduct experiments. HUVECs were treated with varying concentrations of MGO (Sigma-Aldrich, St. Louis, USA) in the presence or absence of 20 mM of l-carnosine (Car) (Sigma-Aldrich) as indicated. The study was approved by local ethics committee (Medizinische Ethik-kommission II der Medizinischen Fakultät Mannheim (No. 2015-518N-MA)) and conducted in accordance with the relevant guidelines and regulations.

### MTT assay

Cell viability was measured by MTT (3-(4, 5-dimethylthiazol-2-yl)-2, 5-diphenyltetrazolium) tetrazolium reduction assay. HUVECs (2.5 × 10^3^ cells/well) were seeded on 96-well plates and cultured in normal medium overnight, followed by treatments with varying concentrations of MGO in absence or presence of 20 mM of carnosine for different time periods. Hereafter, MTT (0.5 mg/ml) in PBS was added to each well and metalized at 37 °C, 5% CO_2_ for 4 h. Crystals of formazan were dissolved by the addition of solvent solution (4 parts DMSO, 4 parts 10% w/v SDS and 2 parts of PBS/acetic acid in a final concentration of 1.2% v/v) and left for overnight incubation at 37 °C, 5% CO_2_. Absorbance was measured with a microplate reader at 560 nm with subtracted background at 670 nm. Relative cell viability was calculated as ratio of OD sample/OD untreated control. For each condition 5 or 6 replicates were used.

### TUNEL assay

TUNEL (Terminal deoxynucleotidyl transferase dUTP nick end labelling) assay was performed using in situ cell death detection kit (Roche, Penzberg, Germany). After treatment with indicated concentrations of MGO for 5 h, HUVECs were fixed in 4% paraformaldehyde, rinsed with PBS, permeabilized in 0.1% Triton X-100, and then processed for TUNEL labelling. Thereafter DAPI (4′, 6-diamidino-2-phenylindole) was stained for nuclear quantitation. Some cells were incubated with DNase I (3000 u/ml) (positive control) prior to labelling procedures to induce DNA strand breaks. After extensive washing, cells were observed under fluorescence microscopy (Axio Observer Z1, Carl Zeiss AG, Germany).

### Immunofluorescence (IF) staining

HUVECs were cultured in the 24-well plates containing glass slides and treated with indicated concentration of MGO for 5 h. Cells were fixed with 4% paraformaldehyde for 15 min and permeabilized with 0.4% Triton for 5 min, prior to the blocking with 1% BSA for 1 h. Hereafter, the specimens were incubated with rabbit-anti-phospho-Histone H2AX (Ser139) (1:200, Cell Signaling Technology) at 4 °C overnight, and then incubated with Alexa Fluor 488-labeled secondary antibody (Thermo Fisher Scientific) at room temperature for 1 h. Immunofluorescence of phospho-Histone H2AX was visualized and assessed under fluorescence microscopy of Leica TCS SP8 (Leica Microsystems, Wetzlar, Germany).

### Quantitative reverse transcription PCR (qRT-PCR)

Total RNA was extracted form HUVECs treated with indicated concentrations of MGO using the of Trizol reagent (Ambion, Carorlsbad, USA). cDNA was generated from 1 µg of RNA following the manual of the High-Capacity cDNA Reverse Transcription Kit (Applied Biosystems, Foster City, USA). Quantitative PCR (qPCR) was performed according to the manual of TaqMan Fast Advanced Master Mix (Applied Biosystems) on a 7900HT Real-Time PCR System (Applied Biosystems). The expression of mRNA targets was normalized to β-actin. The following TaqMan probes (Applied Biosystems) were used: SESN2 (ID: Hs00230241_m1), p21 (ID: Hs00355782_m1), CDK1 (ID: Hs00938777_m1) and β-actin (ID: Hs 01060665_g1). The thermal cycling profile followed by instructions was used: 2 min at 50 °C, 20 s at 95 °C followed by 40 cycles of 1 s at 95 °C and 20 s at 60 °C.

### Western blotting and OxyBlot detection

HUVECs were seeded in 6-well plates and treated with indicated concentrations of MGO in the absence or presence of 20 mM of carnosine at 90%-95% confluence. The whole protein was extracted by using lysis buffer (10 mM Tris, PH 7.4, 150 nM NaCl, 5 mM EDTA, 1% Triton X-100, 0.5% Na-Desoxychalat) supplemented with dithiothreitol (DTT), protease inhibitor and phosphatase inhibitor. Protein concentrations were measured at 595 nm wavelength using Coomassie (Bradford) Protein Assay Kit (Thermo Fisher Scientific). For western blotting, each sample (20 µg) was loaded in 10 or 12% SDS–polyacrylamide gel, followed by gel electrophoresis and semi-dry transfer with Bio-Rad's blotting system. The membrane was blocked with 5% non-fat milk or bovine serum albumin at room temperature for 1 h and cut at appropriate molecular weights of the prestained molecular weight marker (Thermo Fisher Scientific) to allow the use of different antibodies on different parts of the blot. The membrane segments were probed with primary antibodies overnight at 4 °C, followed by incubation with appropriate HRP-conjugated secondary antibodies at room temperature for 1 h. Afterwards, enhanced chemiluminescence (ECL) substrate (Thermo Fisher Scientific) was applied to the membrane. The imaging system of FUSION SL Advance with automatic exposure (Peqlab, Erlangen, Germany) was used to visualize and document chemiluminescence images.

For OxyBlot detection, samples were derivatized following the instructions of OxyBlot Protein Oxidation Detection Kit (Merck Millipore, Darmstadt, Germany), and separated by gel electrophoresis and transferred to a membrane filter as western blotting. The membrane filter was then blocked with 5% non-fat milk or bovine serum albumin at room temperature for 1 h. Afterwards, the membrane was incubated with anti-DNP (1:500–1:1000) overnight at 4 °C and appropriate HRP-conjugated second antibody at room temperature for 1 h. Immunodetection was then performed by using chemiluminescence system of FUSION SL Advance (Peqlab).

### Generation of roGFP3-Grx redox sensor

roGFP3 construct sequence was kind gift from Dr. Manfred Frey. The construct was synthesized by Genewitz (Clone ID B32523-1/q28382) with BamHI and XbaI restriction sites and cloned into SIN lentivirus vector pHR’SIN-cPPT-SEW^61^.

Lentivirus was produced as described in the study of Demaison *et.al.*^62^. Briefly, HEK 293T/17 cells were transiently transfected with the self-inactivating transfer vector plasmid pHR’SIN-roGFP3, pCMV8.91, and pMD.G using METAFECTENE (Biontex Laboratories GmbH, München, Germany). 48 h post transfection the supernatants was collected, filtered and concentrated using Vivaspin 500 centrifugal filter units with MWCO 100,000. The concentrated particles were aliquoted and stored at − 80 °C.

HUVECs were transduced with different concentrations of lentiviral vector for 48 h and transduction efficiency was assessed by visualization of GFP under fluorescence microscope of Leica DFC450 (Leica Microsystems). In transduced HUVECs an optimal expression of GFP was observed at 1:100 dilution and thus this lentiviral concentration was used in further experiments. The stability of the vector over passages was also verified by qualitative assessment of GFP under the florescence microscope. Up to three passages after transduction no evident GFP expression changes were observed.

### Ratiometric measurements

RoGFP3 expressing cells were seeded in 96-well plate. Next day, prior to measurements cell medium was exchange with PBS. We measured the emission of roGFP3 at 528 nm after excitation at 395 and 485 nm in a microplate reader (Tecan, Männedorf, Switzerland) set at 37 °C equipped with built-in injectors. The ratio of emission (395/485 nm) was calculated and plotted it against time. Oxidation of roGFP3-Grx redox sensor resulted in an increase in the emission fluorescence at 528 nm when excited at 485 nm and a decrease in emission fluorescence when excited at 395 nm.

### Statistical analysis

GraphPad Prism 7 (GraphPad Software, Inc. La Jolla, California) was used to perform the statistical analysis. ImageJ program^63^ was applied to quantify images of staining (TUNEL and IF) and densitometry of western blotting. MTT data and quantification of TUNEL and western blotting were analyzed using a one-way analysis of variance (ANOVA) and Tukey’s multiple comparison test. The quantification of IF was analyzed using two-tailed Student’s t-test. Quantitative RT-PCR data were analyzed using a two-way ANOVA and Tukey’s multiple comparison test. Only two-tailed *p*-values less than 0.05 were considered statistically significant.

## Supplementary Information

Supplementary Information.

## Data Availability

All data are available from the authors on reasonable request.
