# Supplementary material for: Methylglyoxal induces p53 activation and inhibits mTORC1 in human umbilical vein endothelial cells
Source: Sci Rep. 2021 Apr 13;11:8004. doi: 10.1038/s41598-021-87561-9 (PMC8044125; doi:10.1038/s41598-021-87561-9)
Supplement: Supplementary file 1 — Supplementary Information. [file 41598_2021_87561_MOESM1_ESM.pdf]

Supplementary information

Methylglyoxal induces p53 activation and inhibits mTORC1 in human umbilical vein endothelial cells

Xinmiao Zhang*[1], Angelica Rodriguez-Niño[1,3], Diego O. Pastene[1], Prama Pallavi[2], Jacob van den Born[3], Stephan J.L. Bakker[3], Bernhard K. Krämer[1,4] and Benito A. Yard[1,4]

[1] Department of Nephrology, Endocrinology and Rheumatology, Fifth Department of Medicine, Medical Faculty Mannheim, Heidelberg University, Mannheim, Germany

[2] Surgical Department, University Hospital Mannheim, Heidelberg University, Mannheim, Germany

[3] Department of Internal Medicine, University Medical Center Groningen, University of Groningen, Groningen, The Netherlands

[4] European Center for Angioscience, Medical Faculty Mannheim, Heidelberg University, Mannheim, Germany

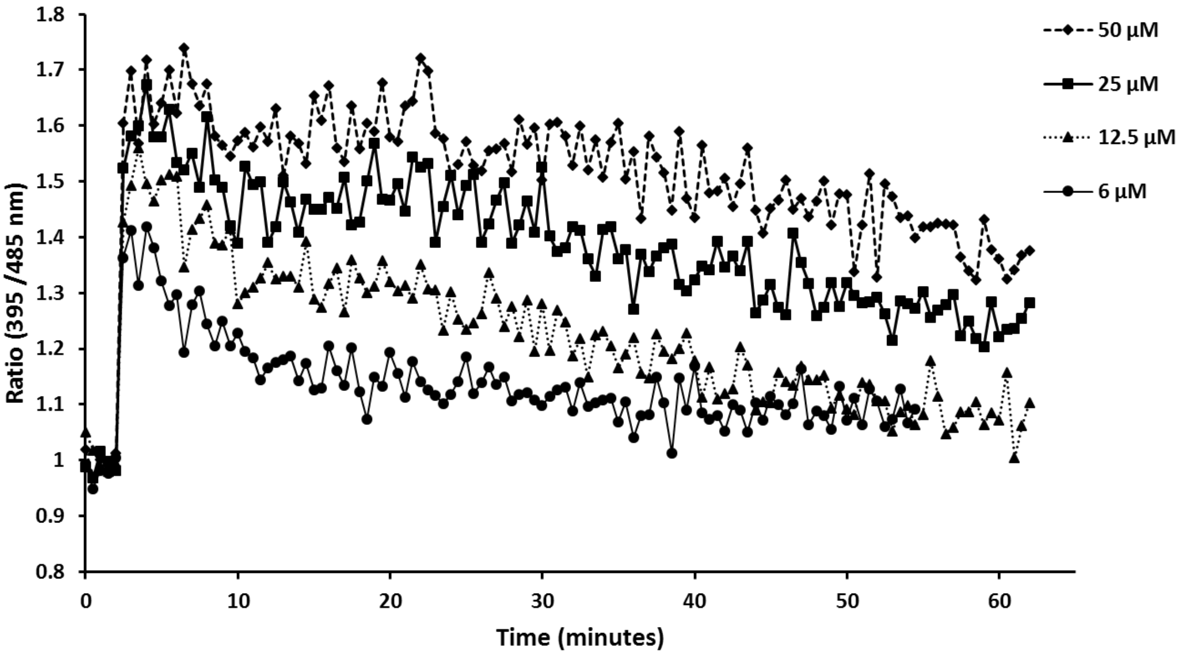

Supplementary Fig. S1: Fluorescence ratio affected by H_2O_2.
In roGFP transduced HUVECs, fluorescence ration at 395/485 nm was increased upon varying concentrations of H_2O_2.

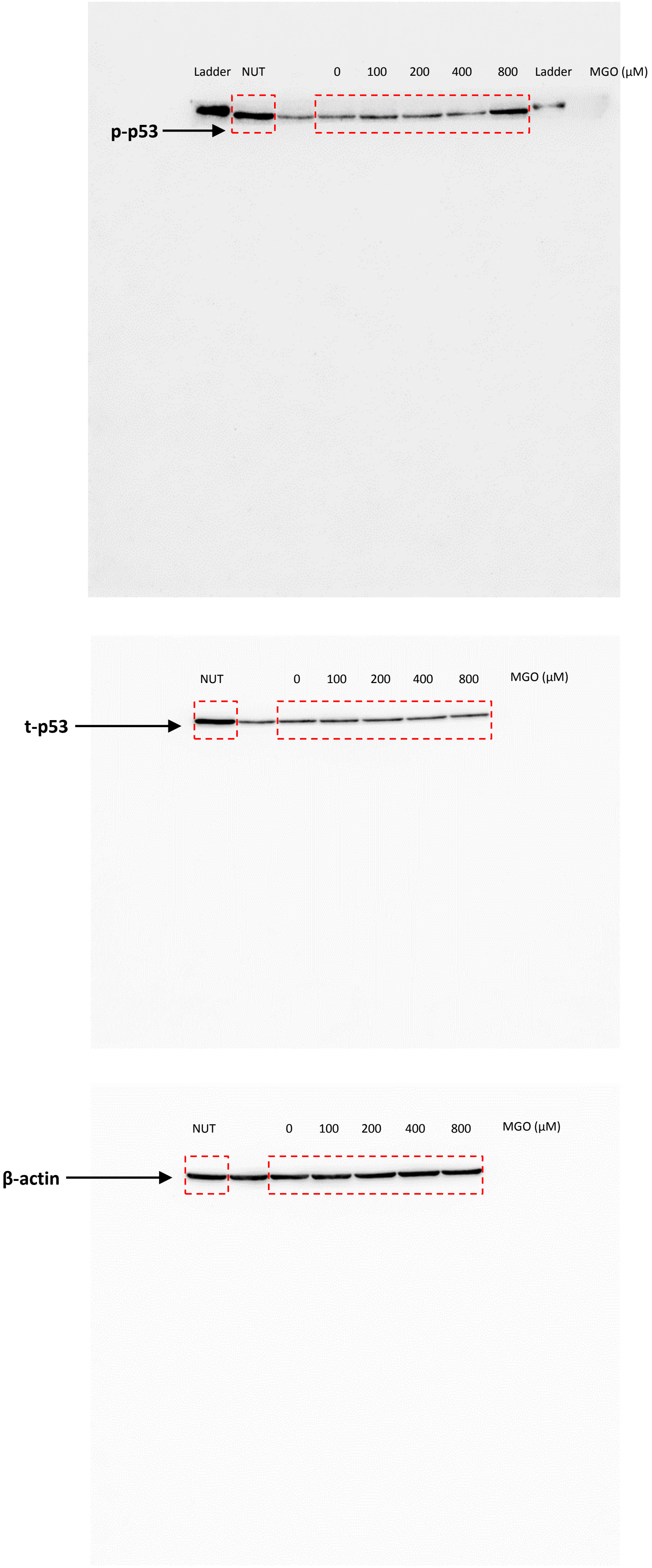

Supplementary Fig. S2: Original blots of Fig. 2A.
Represented are blots cut into segments prior to blotting. The arrows point to the relevant protein bands. Red dashed squares were cropped and shown in Fig. 2A.

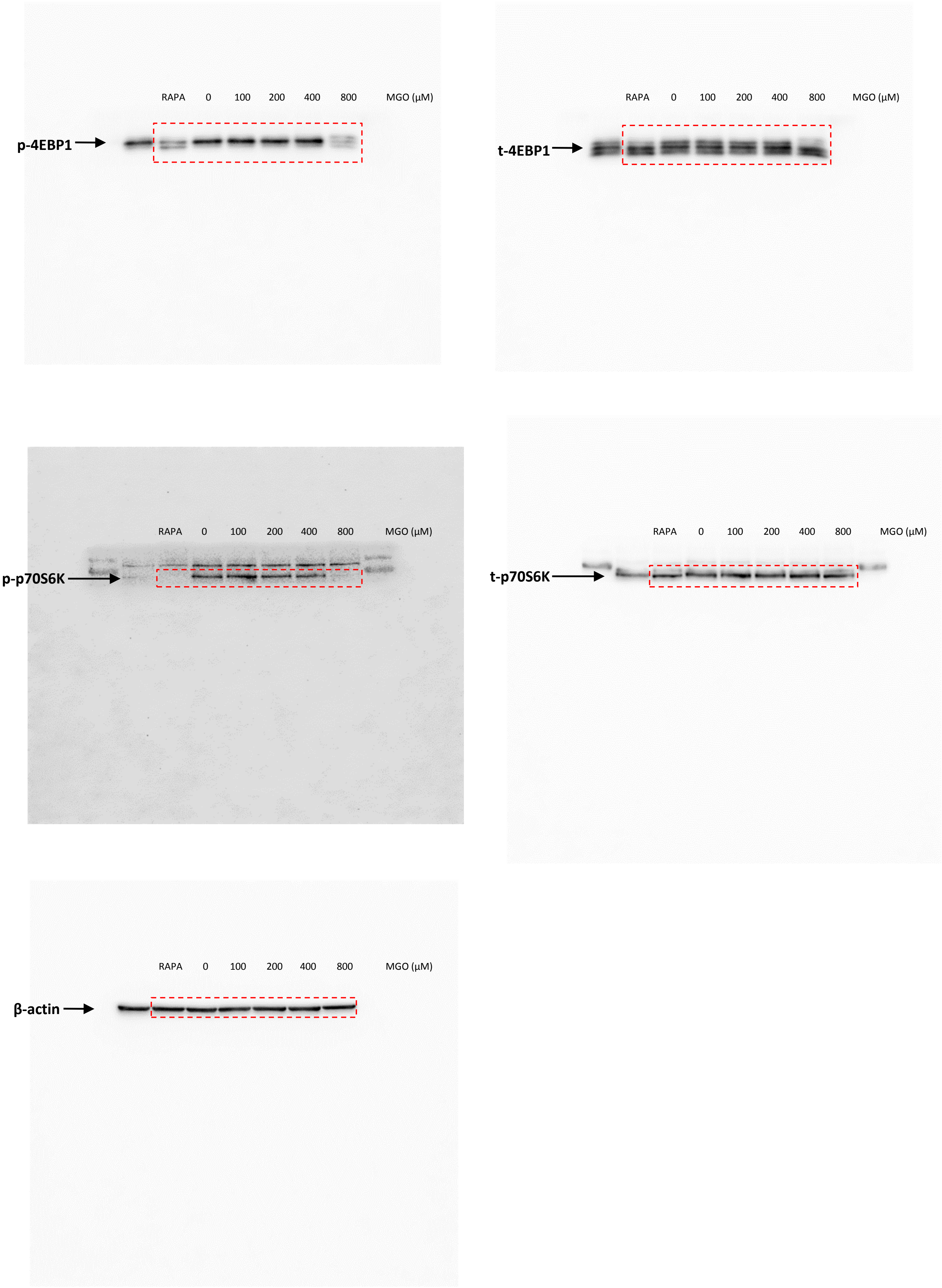

Supplementary Fig. S3: Original blots of Fig. 3A.
Represented are blots cut into segments prior to blotting. The arrows point to the relevant protein bands. Red dashed squares were cropped and shown in Fig. 3A.

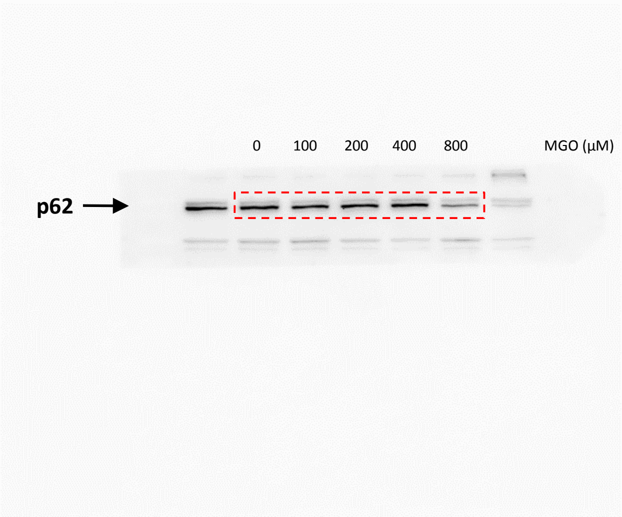

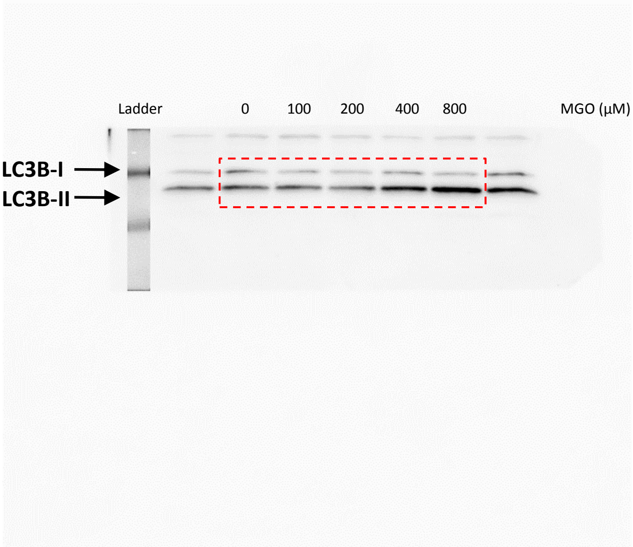

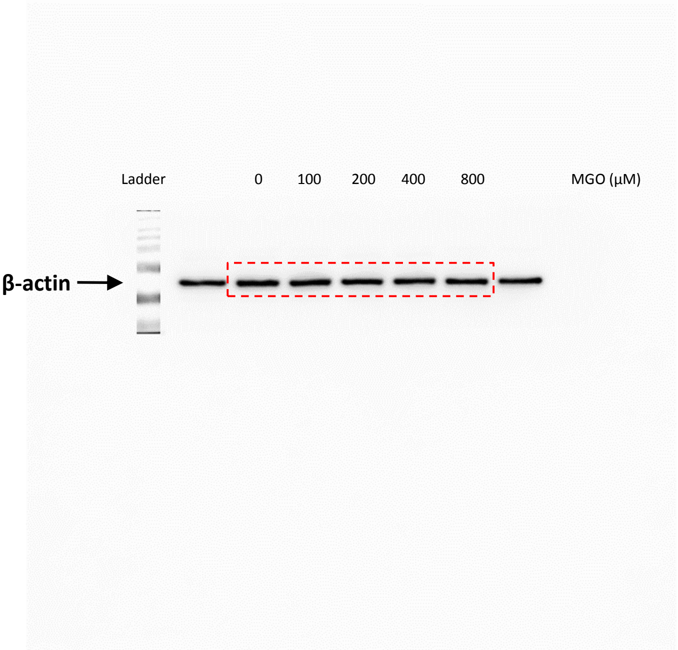

Supplementary Fig. S4: Original blots of Fig. 4A.
Represented are blots cut into segments prior to blotting. The arrows point to the relevant protein bands. Red dashed squares were cropped and shown in Fig. 4A.

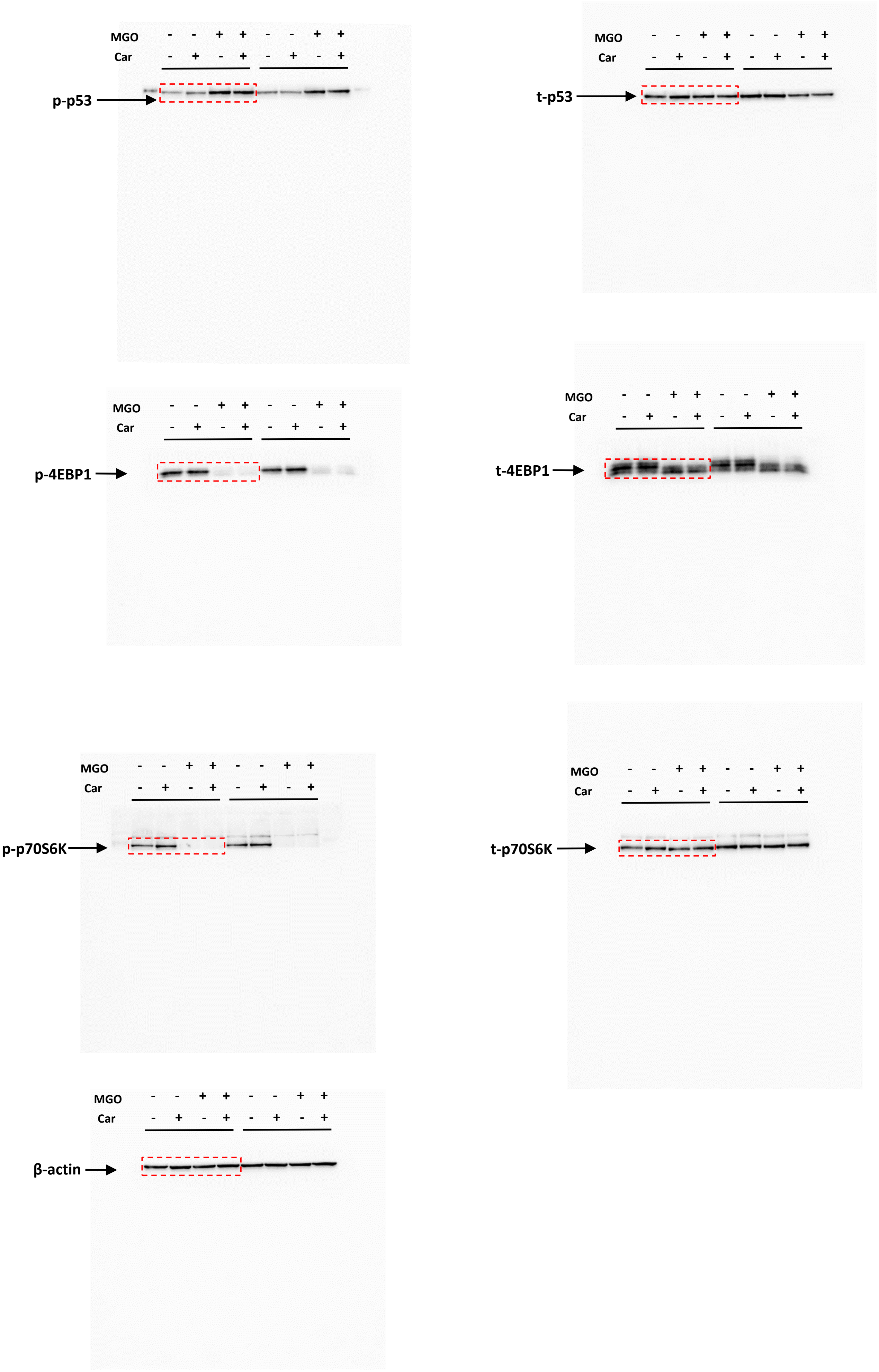

Supplementary Fig. S5: Original blots of Fig. 5A.
Represented are blots cut into segments prior to blotting. The arrows point to the relevant protein bands. Red dashed squares were cropped and shown in Fig. 5A.

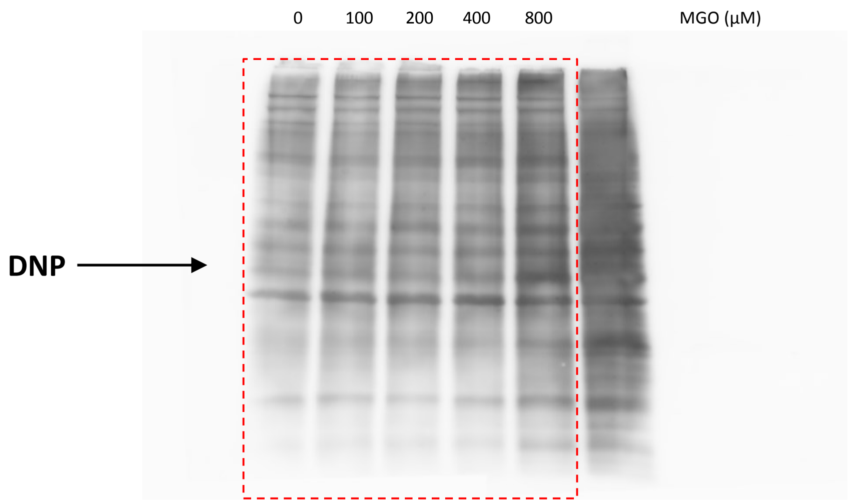

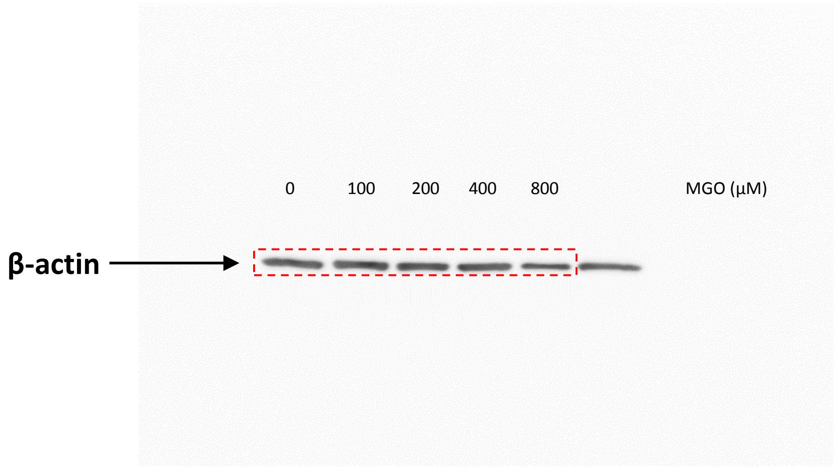

Supplementary Fig. S6: Original blots of Fig. 7A.
The arrows point to the relevant protein bands. Red dashed squares were cropped and shown in Fig. 7A.
